# Association between statin therapy and long-term clinical outcomes in patients with stable coronary disease undergoing percutaneous coronary intervention

**DOI:** 10.1038/s41598-024-63598-4

**Published:** 2024-06-03

**Authors:** Han-Ping Wu, Feng-Ching Yang, Hau-De Lin, Chuan-Zhong Cai, Ming-Jen Chuang, Kuo Feng Chiang, Mao-Jen Lin

**Affiliations:** 1 Department of Pediatrics, Chang-Gung Memorial Hospital, Chiayi County, Taiwan; 2 College of Medicine, Chang Gung University, Taoyuan, Taiwan; 3 Department of Medicine, Taichung Tzu Chi Hospital, The Buddhist Tzu Chi Medical Foundation, Taichung, Taiwan; 4 Department of Medicine, College of Medicine, Tzu Chi University, Hualien, Taiwan; 5 Division of Cardiology, Department of Medicine, Buddhist Taichung Tzu Chi Hospital, 88, Section 1, Fong-Sing Rd, Tanzi District, Taichung City, Taiwan

**Keywords:** Cardiology, Health care

## Abstract

This longitudinal cohort study examined the long-term effect of statin therapy on clinical outcomes in patients undergoing percutaneous coronary intervention (PCI). A total of 1760 patients with stable coronary artery disease (CAD) were divided by receipt of statin therapy or not after index PCI. Baseline clinical characteristics, risk factors, angiographic findings, and medications after interventional procedure were assessed to compare long-term clinical outcomes between groups. Predictors for all-cause death and major adverse cardiovascular events (MACE), including myocardial infarction (MI), cardiovascular death, and repeated PCI procedures, were also analyzed. The statin therapy group had higher average serum cholesterol and more elevated low-density lipoprotein cholesterol (LDL-C) than the non-statin therapy group (189.0 ± 47.9 vs 169.3 ± 37.00 mg/dl, 117.2 ± 42.6 vs 98.7 ± 31.8 mg/dl, respectively, both P < 0.001). The non-statin group had higher rates of all-cause death and cardiovascular death compared to statin group (both P < 0.001). After adjustment for age, diabetes, and chronic kidney disease, Cox proportion hazard analysis revealed statin use significantly reduced all-cause death and repeated PCI procedure (hazard ratio: 0.53 and 0.69, respectively). Statin use seemed not reduce the hazard of cardiovascular death or MI in patients with stable CAD after PCI; however, statin therapy still was associated with reduced rates of all-cause death and repeat PCI procedure.

## Introduction

Stable coronary artery disease (CAD) refers to a reversible supply and demand mismatch related to ischemia, a history of myocardial infarction (MI), or the presence of plaque documented by catheterization or computed tomography angiography. Patients are considered stable if they are asymptomatic or their symptoms are controlled by medication or revascularization^1^. Percutaneous coronary intervention (PCI) is an important therapeutic strategy for coronary revascularization in patients with CAD, and pharmacologic treatment is often necessary to modify risk factors. Among these, dyslipidemia is a key risk factor for coronary atherosclerosis, and also affects outcomes in CAD patients undergoing PCI^2^.

Evidence-based medicine suggests that the use of statins to treat hypercholesterolemia can improve the prognosis of those with cardiovascular (CV) disease^3,4^. In hypertensive patients with normal serum cholesterol levels, statins can also effectively reduce the incidence of future CV events^5^. On the other hand, the prognosis for patients with stable CAD who receive intensive medical therapy, including statins, does not differ from that of patients who receive coronary intervention^6^. Nevertheless**,** for the short-term outcome of acute coronary syndrome, lipid-lowering therapy with statins could reduce recurrent ischemic events in the first 16 weeks^7^; for patients with acute coronary syndrome undergoing coronary intervention, the use of intensive statin therapy before and after cardiac catheterization can reduce the incidence of adverse CV events within 30 days^8,9^. Some studies have reported that, in patients with stable CAD, compared to low-dose or moderate-intensity statin therapy, high-dose or high intensity statin use can further improve CV outcomes^10–12^.

However, in patients with CAD undergoing coronary intervention, the use of statins before cardiac catheterization can reduce the risk of myocardial injury during coronary intervention^13^. Nevertheless, in terms of long-term clinical outcomes in patients with stable CAD undergoing PCI, it remains unclear whether patients with average low-density lipoprotein-cholesterol (LDL-C) levels without statin treatment differ from those with elevated LDL-C levels receiving statin treatment. Therefore, the purpose of the current study is to compare long-term outcomes by statin use in patients undergoing PCI. In addition, predictors of clinical outcomes in stable CAD patients undergoing PCI were also analyzed.

## Methods

### Study population

This retrospective cohort study was conducted from 2012 through 2020. We recruited patients with stable CAD aged between 20 and 85 years from the Inpatient Department of Taichung Tzu Chi Hospital, Taiwan. According to the guideline provided by the Taiwan National Health Insurance Administration, Ministry of Health and Welfare, statins were prescribed in CAD patients with an LDL-C level above 100 mg/dL. Patient who received index PCI with a serum LDL-C above 100 mg/dl and were statin-naïve were considered as receiving statin therapy; those receiving statin therapy prior to index PCI were excluded. All patients were divided into two groups: non-statin users and statin users. Patients with scheduled PCI, New York Heart Association (NYHA) class IV heart failure, and the presence of malignancy were excluded from this study. The majority of patients received regular follow up via the outpatient department. The very few patients lost to follow up were called by telephone to attempt contact. The study was approved by the Institutional Review Board of Taichung Tzu Chi Hospital (REC111-62), and informed consent were obtained from all participants. This cohort study was performed in accordance with the relevant guidelines and regulations, and also fulfilled the guidance of the Strengthening the Reporting of Observational Studies in Epidemiology (STROBE) statement^14^.

### Data gathering and analysis

Data pooled included general characteristics such as age, gender, and biochemical profiles, including serum levels of total cholesterol, high-density lipoprotein cholesterol (HDL-C), LDL-C, and triglycerides. Angiographic findings during cardiac catheterization were recorded, including lesion location and the number of diseased vessels and lesions. Lesion severity and complexity were calculated via the Synergy between PCI with Taxus and cardiac surgery score (SYNTAX Score)^15^. Left ventricular systolic function was evaluated as ejection fraction through contrast ventriculography or nuclear ventriculography. Use of interventional procedures such as balloon angioplasty, bare metal stent, or drug-eluting stent (DES) was also analyzed. The types and dosages of statins used in this study was also fully surveyed. The previously-stated definitions of major risk factors were used^16–20^. General characteristics, risk factors, angiographic findings, and types of PCI strategies were compared. The clinical end-points were all-cause death and major adverse cardiovascular events (MACE), including CV mortality, MI, and clinically-driven repeated PCI procedures. The beginning of follow up was the date of the index PCI procedure, and the duration of follow up was from the index date through December 2021 or the occurrence of any of the above primary end-points.

### Statistical analysis

The analysis was used primarily to test differences between groups. Given a type I (a) error of 0.05, type II (b) error of 0.2, the proportion of stain among all subjects of 0.39, detectable OR of 1.8, and we assume the prevalence of stable CAD is 8%, the minimum sample size required for the stable CAD under statin therapy group is 215. Independent t-test was used to examine numerical variables for statistically significant differences between the means in non-statin group and statin group. Chi-square test or Fisher-exact test, as appropriate, was used to examine categorical variables. Log-rank tests and Kaplan–Meier curves were used to compare survival differences. The Cox proportional regression model was used to test the effect of independent variables on hazards, and hazard ratio (HR) and 95% confidence interval (CI) were used to describe the relative risk. A P value of less than 0.05 was considered significant. All analyses were performed using the statistical package SPSS for Windows, Version 25.0 (IBM Corp., Armonk, NY, USA).

## Results

**Figure 1:**
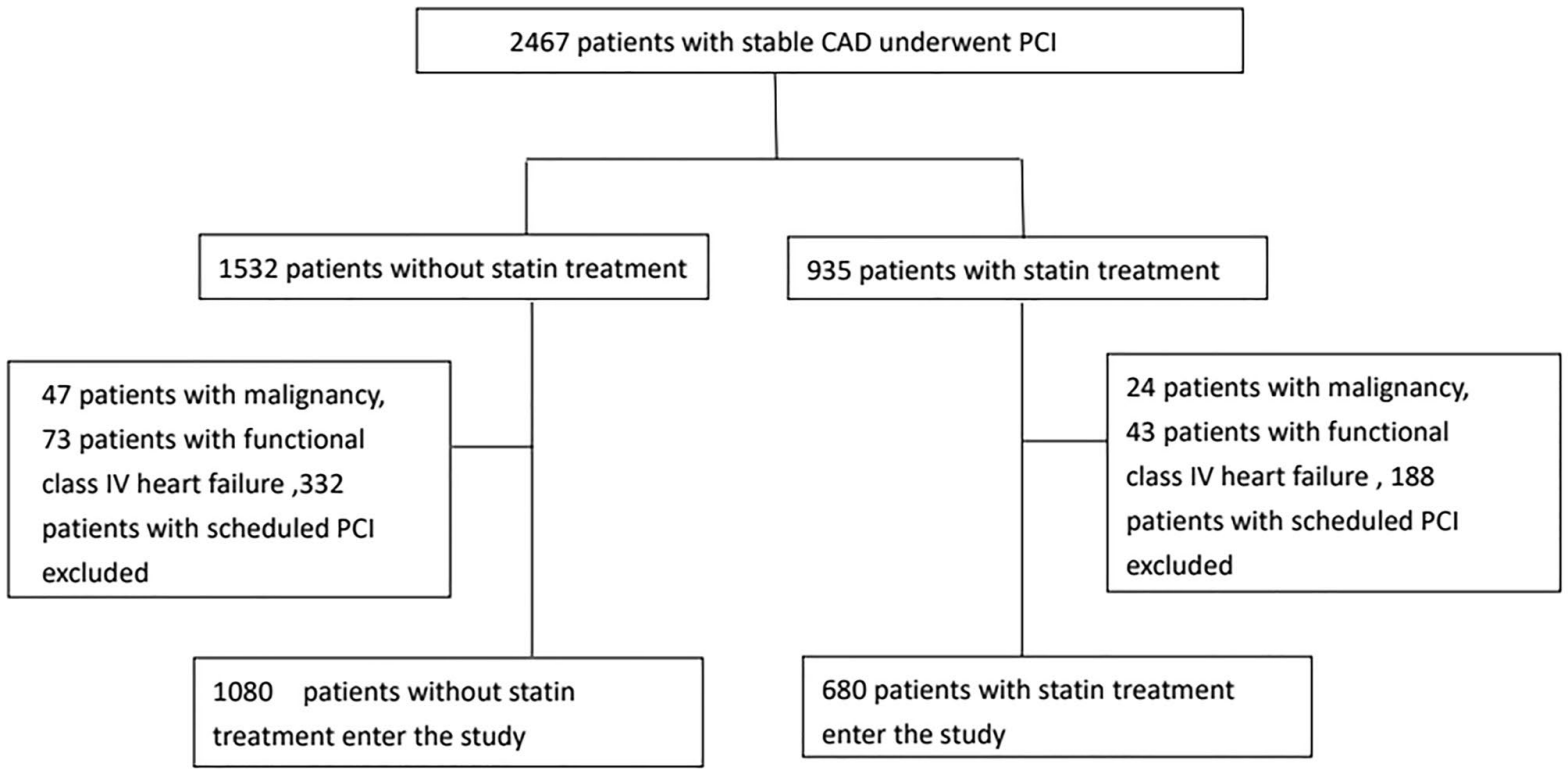
Algorithm for study population enrollment.

During the 4-year study period, a total of 1760 patients with stable CAD who underwent a successful PCI procedure were enrolled. The algorithm for enrollment of the study population is shown in Fig. 1. Among them, 1080 patients were in the non-statin users group (102 patients withdrew from statin use due to intolerance within 3 months after index PCI), while 680 patients were in the statin users group. In the statin users group, 365 patients used rosuvastatin (53.7%, median dose: 10 mg), 239 patients used atorvastatin (35.1%, median dose: 20 mg), 44 patients used simvastatin (6.5%, median dose: 20 mg), and 32 patients used pitavastatin (4.7%, median dose: 2 mg). Statins were prescribed after index PCI for one year or longer. The follow-up time for the non-statin users group was 45.2 ± 31.7 months versus 44.4 ± 36.1 months for the statin users group v (P = 0.61).

General characteristics of the study groups are listed in Table 1. Statin users were younger than non-statin users (62.1 ± 11.0 vs 65.2 ± 11.4 years, P < 0.01) and also had a lower serum creatinine level than non-statin therapy patients (1.4 ± 3.8 vs 1.9 ± 2.4 mg/d1, P < 0.01).As for baseline biochemistry, the statin user group had higher average total cholesterol (189.0 ± 47.9 vs 169.3 ± 37.0 mg/dL) and elevated LDL-C levels (117.2 ± 42.6 vs 98.7 ± 31.8 mg/dL) than the non-statin user group (both P < 0.01).

**Table 1:** General characteristics of the study population between groups.

Variable	Study groups		P value
	Statin (N = 680)	Non-Statin (N = 1080)	
Age (years)	62.1 ± 11.0	65.2 ± 11.4	< 0.01*
Weight (kg)	69.9 ± 12.2	67.9 ± 12.3	< 0.01*
Height (m)	1.63 ± 0.08	1.62 ± 0.08	< 0.01*
BMI (kg/m^2^)	26.1 ± 3.9	25.8 ± 3.9	0.07
CSP (mmHg)	138.6 ± 21.8	138.8 ± 22.6	0.85
CDP (mmHg)	74.5 ± 12.3	73.4 ± 12.3	0.06
CPP (mmHg)	64.1 ± 19.6	65.3 ± 20.9	0.23
Cholesterol (mg/dl)	189.0 ± 47.9	169.3 ± 37.0	< 0.01*
HDL (mg/dl)	38.3 ± 16.9	39.6 ± 15.9	0.11
TG (mg/dl)	167.3 ± 104.0	155.1 ± 109.4	0.02*
LDL (mg/dl)	117.2 ± 42.6	98.7 ± 31.8	< 0.01*
Creatinine (mg/dl)	1.4 ± 3.8	1.9 ± 2.4	< 0.01*

BMI: body mass index. CSP: central aortic systolic pressure. CDP: central aortic diastolic pressure. CPP: central aortic pressure. HDL: high-density lipoprotein cholesterol. LDL: low-density lipoprotein cholesterol. TG: triglyceride.

*Significant.

The demographic and clinical profiles of the study population are shown on Table 2. There was no gender difference between the two groups. The non-statin user group had a higher prevalence of diabetes mellitus (DM) and chronic kidney disease (CKD) than the statin user group (both P < 0.01). In terms of prescribed medication after index PCI, the statin user group used aspirin, P2Y12 inhibitor, and angiotensin receptor blocker more frequently (P < 0.01, P = 0.01, P < 0.01, respectively); however, the non-statin user group had a higher rate of diuretics and fibrate usage (P = 0.03, P < 0.01, respectively).

**Table 2:** Demographics of the study population, and medications prescribed after index PCI between groups.

Variable	Study groups		P value
	Statin (N = 680)	Non-Statin (N = 1080)	
Gender			0.85
F	179 (26.3%)	280 (25.9%)	
M	501 (73.7%)	800 (74.1%)	
Hypertension			0.34
No	281 (41.3%)	471 (43.6%)	
Yes	399 (58.7%)	609 (56.4%)	
DM			< 0.01*
No	451 (66.3%)	635 (58.8%)	
Yes	229 (33.7%)	445 (41.2%)	
CKD			< 0.01*
No	479 (70.4%)	585 (54.2%)	
Yes	201 (29.6%)	495 (45.8%)	
Stroke history			0.51
No	646 (95.0%)	1018 (94.3%)	
Yes	34 (5.0%)	62 (5.7%)	
CABG history			0.05
No	671 (98.7%)	1075 (99.5%)	
Yes	9 (1.3%)	5 (0.5%)	
Aspirin			< 0.01*
No	49 (7.2%)	137 (12.7%)	
Yes	631 (92.8%)	943 (87.3%)	
P2Y12 inhibitors			0.01*
No	94 (13.8%)	200 (18.5%)	
Yes	586 (86.2%)	880 (81.5%)	
Diuretics			0.03*
No	578 (85.0%)	874 (80.9%)	
Yes	102 (15.0%)	206 (19.1%)	
BB			0.36
No	376 (55.3%)	621 (57.5%)	
Yes	304 (44.7%)	459 (42.5%)	
CCB			0.07
No	439 (64.6%)	651 (60.3%)	
Yes	241 (35.4%)	429 (39.7%)	
ACEI			0.90
No	612 (90.0%)	970 (89.8%)	
Yes	68 (10.0%)	110 (10.2%)	
ARB			< 0.01*
No	441 (65.0%)	780 (72.2%)	
Yes	237 (35.0%)	300 (27.8%)	
Fibrate			< 0.01*
No	672 (98.8%)	979 (90.7%)	
Yes	8 (1.2%)	101 (9.4%)	

DM: diabetes Mellitus. CKD : chronic kidney disease alone. CABG history: history of coronary artery bypass graft. CKD: chronic kidney disease. P2Y12 inhibitor: P2Y12 receptor inhibitor of platelet. BB: beta-blockers. CCB: calcium channel blocker. ACEI: angiotensin-converting enzyme inhibitor. ARB: angiotensin receptor blocker.

*Significant.

**Figure 2:**
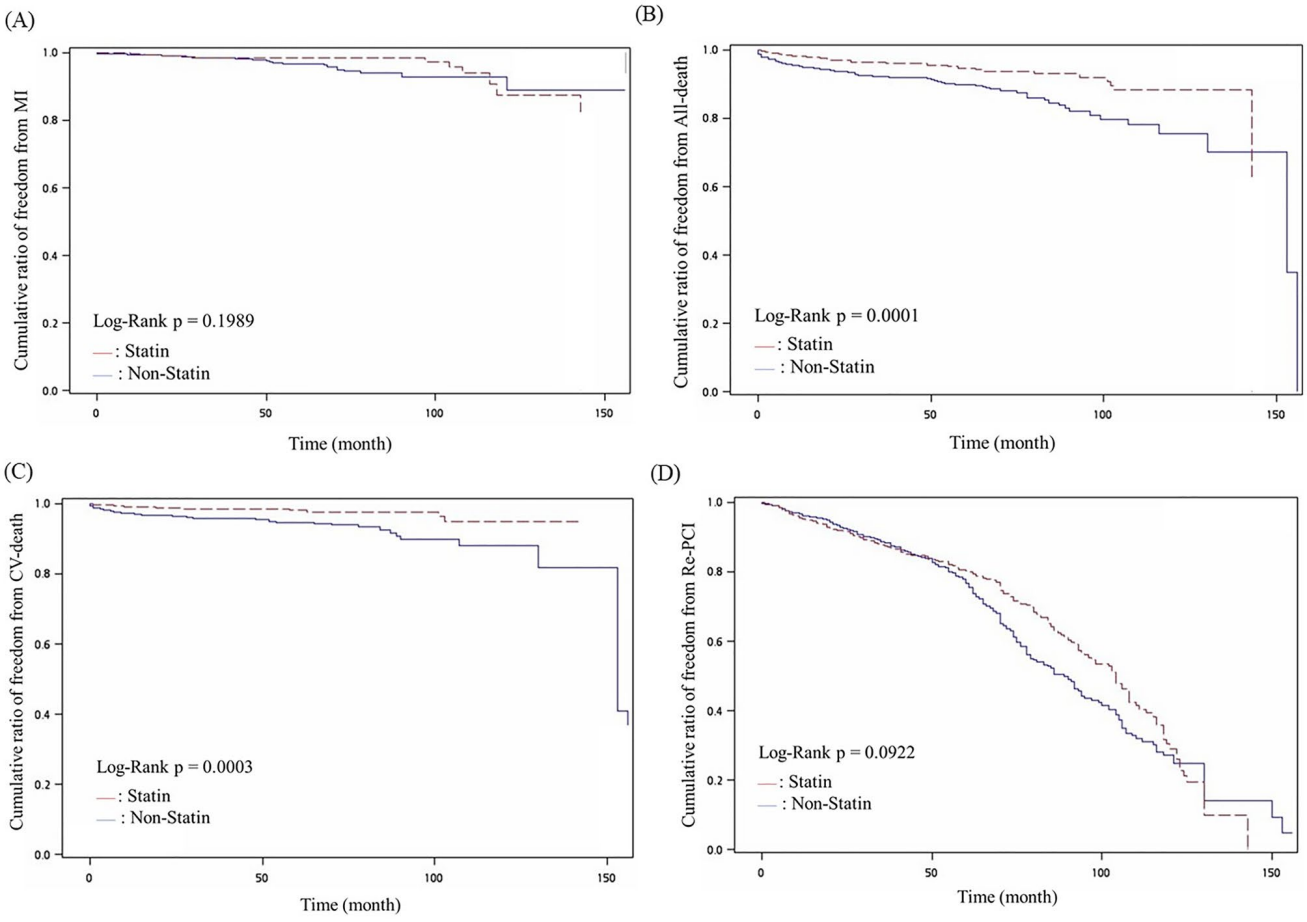
Kaplan–Meier survival curves for statin users and non-users. (**A**) Cumulative ratio of myocardial infarction (MI) between groups (P = 0.1989). (**B**) Cumulative ratio of all-cause death between groups (P < 0.001). (**C**) Cumulative ratio of cardiovascular (CV) death between groups (P < 0.001). (**D**) Cumulative ratio of repeated percutaneous coronary intervention (PCI) between groups (P = 0.0922).

Results of the angiographic findings and clinical endpoints are shown in Table 3. The angiographic findings revealed no significant difference between groups in the number of diseased vessels, treated vessels, or lesions (P = 0.61). However, the statin user group had a higher SYNTAX score than the non-statin user group (P < 0.01). The type of intervention did not differ between groups. Rates of CV death and all-cause death were higher in the non-statin user group than in the statin user group (both P < 0.01). Figure 2 reveals the cumulated rates of MI, CV death, all-cause death, and clinically-driven repeated PCI procedures for the two groups. The Kaplan–Meier survival curves indicate that the statin user group had lower rates of all-cause death and CV death (both P < 0.001), but the groups did not differ in rates of MI (P = 0.20) or clinically-driven repeated PCI procedures (P = 0.09).

**Table 3:** Demography of angiographic findings and outcome between groups.

Variable	Study groups		P value
	Statin (N = 680)	Non-Statin (N = 1080)	
Follow-up time (months)	44.4 ± 36.1	45.2 ± 31.7	0.61
Number of diseased vessels			0.37
Single vessel disease	345 (50.7%)	582 (53.9%)	
Dual vessel disease	194 (28.5%)	298 (27.6%)	
Triple vessel disease	141 (20.7%)	200 (18.5%)	
Mean of treated vessels	1.7 ± 0.8	1.6 ± 0.8	0.16
Mean of treated lesions	1.3 ± 0.5	1.3 ± 0.5	0.07
SYNTAX score	10.3 ± 7.4	9.2 ± 7.0	< 0.01*
LVEF	0.59 ± 0.13	0.60 ± 0.13	0.03
CPP	64.1 ± 19.6	65.3 ± 20.9	0.23
Type of intervention			0.07
Balloon angioplasty	180 (26.5%)	350 (32.4%)	
BMS deployment	221 (32.5%)	347 (32.1%)	
DES deployment	378 (55.6%)	513 (47.5%)	
MI			0.37
Yes	13 (1.9%)	31 (2.9%)	
No	677 (98.1%)	1049 (97.1%)	
CV death			< 0.01*
Yes	12 (1.8%)	56 (5.2%)	
No	668 (98.2%)	1024 (94.8%)	
All-cause death			< 0.01*
Yes	32 (4.7%)	107 (9.9%)	
No	648 (95.3%)	973 (90.1%)	
Repeated-PCI			0.54
Yes	176 (25.9%)	294 (27.2%)	
No	504 (74.1%)	786 (72.8%)	

BMS: bare metal stent. CPP: central pulse pressure ,equal to central systolic pressure minus central aortic pressure. DES: drug-eluting stent. SYNTAX score: Synergy between Percutaneous Coronary Intervention with Taxus and Cardiac Surgery score. LVEF: left ventricular ejection fraction. MI: myocardial infarction. Repeated-PCI: repeated percutaneous coronary intervention.

*Significant.

Table 4 shows the main results of an outcome analysis based on a Cox proportion regression model. Briefly, SYNTAX score predicted MI, after adjustment for relevant variables. After adjustment, DM and CKD predicted all-cause death, while use of statins, beta blockers (BB), calcium channel blockers (CCB), and angiotensin converting enzyme inhibitors (ACEI) reduced the rate of all-cause death, after adjustment. P2Y12 inhibitor usage increase the rate of CV death after adjustment, while aspirin usage and DES deployment reduced the rate of CV death, after adjustment. Finally, the presence of CKD and usage of P2Y12 inhibitor predicted clinically-driven repeated PCI procedures after adjustment, while usage of ACEI and statins reduced the rate of clinically-driven repeated PCI procedures, after adjustment.

**Table 4:** Significant predictors of outcome for MI, All-cause death, CV-death, and Repeated PCI in Cox proportion hazard model.

Crude HR (95%C.I.)	MI			All-cause death		CV-death		Repeated PCI	
	Crude HR (95%C.I.)		Adjusted HR (95%C.I.)^a^	Crude HR (95%C.I.)	Adjusted HR (95%C.I.)^a^	Crude HR (95%C.I.)	Adjusted HR (95%C.I.)^a^	Crude HR (95%C.I.)	Adjusted HR (95%C.I.)^a^
Age	1.00 (0.96–1.04)		1.00 (0.95–1.04)	1.01 (0.99–1.04)	1.01 (0.99–1.04)	1.01 (0.98–1.04)	0.98 (0.94–1.01)	0.99 (0.98–1.00)	0.99 (0.98–1.00)
DM	1.45 (0.66–3.21)		1.95 (0.81–4.70)	1.24 (0.78–1.97)	1.91 (1.17–3.13)**	0.81 (0.42–1.59)	0.83 (0.35–1.98)	1.00 (0.78–1.28)	0.98 (0.77–1.26)
CKD	1.94 (0.80–4.71)		1.30 (0.52–3.29)	3.01 (1.74–5.20)**	2.09 (1.21–3.63)**	3.28 (1.50–7.20)**	1.18 (0.51–2.70)	1.53 (1.17–2.00)**	1.44 (1.10–1.90)**
Stroke	0.72 (0.10–5.48)		0.71 (0.09–5.46)	1.36 (0.58–3.18)	1.53 (0.64–3.65)	1.52 (0.46–5.07)	1.56 (0.39–6.26)	0.90 (0.49–1.67)	0.97 (0.53–1.79)
Syntax score	1.04 (0.99–1.09)		1.04 (1.00–1.09)*	0.99 (0.95–1.02)	0.99 (0.96–1.02)	0.99 (0.94–1.04)	1.02 (0.97–1.08)	1.01 (0.99–1.02)	1.01 (0.99–1.02)
DES	0.73 (0.31–1.73)		0.80 (0.32–1.98)	0.68 (0.40–1.17)	1.00 (0.55–1.83)	0.40 (0.18–0.89)*	0.35 (0.13–0.93)*	1.06 (0.82–1.38)	1.08 (0.83–1.40)
Aspirin	2.89 (0.39–21.62)		2.07 (0.27–15.98)	2.05 (0.82–5.12)	2.01 (0.76–5.31)	1.20 (0.42–3.44)	0.18 (0.05–0.73)*	1.61 (0.96–2.68)	1.52 (0.91–2.54)
P2Y12 inh	1.50 (0.53–4.29)		1.84 (0.61–5.54)	1.28 (0.72–2.26)	0.89 (0.49–1.64)	2.44 (0.99–6.01)	4.89 (1.55–15.46)**	1.44 (1.04–1.99)*	1.52 (1.09–2.12)*
BB	1.20 (0.55–2.60)		1.00 (0.43–2.31)	0.52 (0.31–0.85)**	0.34 (0.20–0.60)**	0.69 (0.35–1.36)	1.24 (0.50–3.07)	1.02 (0.81–1.30)	1.03 (0.81–1.31)
CCB	0.86 (0.39–1.90)		0.79 (0.33–1.88)	0.55 (0.33–0.90)*	0.58 (0.34–0.99)*	0.65 (0.32–1.29)	1.50 (0.60–3.72)	0.98 (0.77–1.24)	1.03 (0.81–1.32)
ACEI	0.50 (0.17–1.53)		0.33 (0.10–1.05)	0.82 (0.46–1.47)	0.49 (0.26–0.93)*	1.26 (0.60–2.67)	2.34 (0.82–6.70)	0.73 (0.54–0.98)*	0.72 (0.54–0.98)*
Statin	0.63 (0.26–1.49)		0.78 (0.31–1.93)	0.54 (0.31–0.94)*	0.53 (0.30–0.94)*	0.51 (0.23–1.14)	1.02 (0.40–2.57)	0.70 (0.55–0.90)**	0.69 (0.53–0.89)**

DM: diabetes Mellitus. CKD: chronic kidney disease. SYNTAX score: Synergy between Percutaneous Coronary Intervention with Taxus and Cardiac Surgery score.DES: drug-eluting stent. P2Y12 inh: P2Y12 receptor inhibitor of platelet. BB: beta-blockers. CCB: calcium channel blocker. ACEI: angiotensin-converting enzyme inhibitor.

^a^Adjusted for MI, all-death, CV-death, and repeated PCI. *P < 0.05, **P < 0.01.

## Discussion

In the current study, statin usage in patients with stable CAD seemed to be associated with reduction in the rates of all-cause death and clinically-driven repeated PCI procedures; however, statin usage did not reduce the rates of MI or CV death. In addition, we found that the SYNTAX score was a predictor for MI. Presence of DM and CKD were both predictors of all-cause death, while usage of BB, CCB, ACEI, and statins could reduce the risk of all-cause death. P2Y12 inhibitor usage was a predictor for CV death, while aspirin and DES deployment reduced CV death. The presence of CKD and usage of P2Y12 inhibitor predicted repeated PCI procedures, while usage of ACEI and statins could reduce the risk of repeated PCI procedures.

In patients with stable CAD undergoing PCI, there was no gender difference by statin use, but the average age of those in the non-statin user group was older than that of the statin user group. The average serum cholesterol level was higher in the statin user group than in the non-statin user group. The average serum LDL-C level in the statin user group was higher than 100 mg/dL, whereas that in the non-statin user group was lower than 100 mg/dL, results in line with guideline-recommend medical therapy (GRMT)^17,18^. In the non-statin user group, 102 patients withdrew from statin therapy within 3 months after PCI, mainly due to statin intolerance, such as severe myalgia, elevated creatine kinase, and threefold elevation of baseline aspartate aminotransferase and alanine aminotransferase. In patients with acute coronary syndrome with an LDL level of 50–125 mg/dL, statin treatment has been proven to improve CV outcomes^21^. In the current study, statin usage seemed to be beneficial in patients with stable CAD receiving PCI with an average serum LDL-C above 100 mg/dl; however; its usefulness in those with serum LDL-C levels below 100 mg/dl remains unclear.

Compared with the statin user group, those in the non-statin user group had higher rates of DM and CKD, which might imply that the non-statin user group had more risk factors. In patients undergoing PCI procedures, DM and CKD negatively impact long-term outcomes^22,23^. In the current study, the presence of DM or CKD was related to the rate of all-cause death; the presence of CKD was also related to an increased rate of repeated PCI procedures in all patients. Previous literature stated a combination of insulin resistance and endothelial dysfunction leads to the progression of atherosclerosis in patients with DM or CKD^24,25^, which might increase the incidence of mortality and morbidity. As for medications prescribed after PCI, aspirin was prescribed less frequently in the non-statin group than in the statin group. The greater age of the non-statin group may have caused aspirin to be underused due to intolerance and increased risk of bleeding. Furthermore, dual-antiplatelet therapy is not superior to aspirin alone in patients at high risk for atherothrombotic events^26^. Given the interventional strategy, the use of drug-eluting stents seemed to reduce the rate of CV death in the current study; nevertheless, a post-hoc analysis revealed that patients with stable CAD remain at substantial risk for long-term MACE after revascularization with PCI with contemporary DES^27^.

Although the non-statin group was older than the statin group and lead-time bias may exist, statin therapy still had a positive role in reducing the rate of all-cause death and repeated PCI procedures after adjustment for age, DM, and CKD in the present study, results compatible with a study conducted in Japan^28^. In addition to improving coronary microvascular dysfunction and reducing MACE in patients receiving coronary angiography, long-term statin therapy also has been shown to improve epicardial coronary perfusion after PCI^29^. Statin therapy has also been associated with a low rate of all-cause mortality in patients with non-obstructive CAD^30^.

Several limitations exist in this study. First, we did not survey the intensity of medical control for lipids, blood glucose, or blood pressure. Second, since the majority of statin users in the current study received rosuvastatin or atorvastatin, the impact of simvastatin and pitavastatin on long-term outcomes is obscure. Third, since both groups had too few MIs to yield statistical significance, the possibility of inadequate participants and follow-up time cannot be excluded. Fourth, since the analysis of the current study was based on GRMT, whether statin usage could improve long-term outcomes in stable CAD patients undergoing PCI with a serum LDL-C level less than 100 mg/dL remains to be determined.

## Conclusion

In patients with stable CAD undergoing PCI with mildly elevated serum LDL-C levels, statin usage may not reduce CV mortality. Nevertheless, statin use was still associated with a reduction in the rate of all-cause mortality and clinically-driven repeated PCI procedures.

## Data Availability

The data that support the findings of this study are available from the corresponding author on reasonable request.

## Abbreviations

ACEI: Angiotensin-converting enzyme inhibitor
BB: Beta blockers
CAD: Coronary artery disease
CCB: Calcium channel blockers
CKD: Chronic kidney disease
CV: Cardiovascular
DES: Drug-eluting stent
GRMT: Guideline recommend medical therapy
DM: Diabetes mellitus
HDL-C: High-density lipoprotein-cholesterol
LDL-C: Low-density lipoprotein-cholesterol
MACE: Major adverse cardiovascular events
MI: Myocardial infarction
PCI: Percutaneous coronary intervention
STROBE: Strengthening the Reporting of Observational Studies in Epidemiology
SYNTAX score: Synergy between Percutaneous Coronary Intervention with Taxus and Cardiac Surgery score
